# Effects of alcohol on the composition and metabolism of the intestinal microbiota among people with HIV: A cross-sectional study

**DOI:** 10.1016/j.alcohol.2024.02.003

**Published:** 2024-11

**Authors:** Ni-ni Qiao, Quan Fang, Xin-hong Zhang, Su-su Ke, Zi-wei Wang, Gan Tang, Rui-xue Leng, Yin-guang Fan

**Affiliations:** Department of Epidemiology and Health Statistics, Anhui Medical University, Hefei, 230032, Anhui, People's Republic of China

**Keywords:** alcohol, cross-sectional study, HIV/AIDS, intestinal microbiota, metabolism

## Abstract

**Objectives:**

Alcohol consumption is not uncommon among people with HIV (PWH) and may exacerbate HIV-induced intestinal damage, and further lead to dysbiosis and increased intestinal permeability. This study aimed to determine the changes in the fecal microbiota and its association with alcohol consumption in HIV-infected patients.

**Methods:**

A cross-sectional survey was conducted between November 2021 and May 2022, and 93 participants were recruited. To investigate the alterations of alcohol misuse on fecal microbiology in HIV-infected individuals, we performed 16s rDNA gene sequencing on fecal samples from the low-to-moderate drinking (n = 21) and non-drinking (n = 72) groups.

**Results:**

Comparison between groups using alpha and beta diversity showed that the diversity of stool microbiota in the low-to-moderate drinking group did not differ from that of the non-drinking group (all *p* > 0.05). The Linear discriminant Analysis effect size (LEfSe) algorithm was used to determine the bacterial taxa associated with alcohol consumption, and the results showed altered fecal bacterial composition in HIV-infected patients who consumed alcohol; *Coprobacillus*, *Pseudobutyrivibrio*, and *Peptostreptococcaceae* were enriched, and *Pasteurellaceae* and *Xanthomonadaceae* were depleted. In addition, by using the Kyoto Encyclopedia of Genes and Genomes (KEGG), functional microbiome features were also found to be altered in the low-to-moderate drinking group compared to the control group, showing a reduction in metabolic pathways (*p* = 0.036) and cardiovascular disease pathways (*p* = 0.006).

**Conclusion:**

Low-to-moderate drinking will change the composition, metabolism, and cardiovascular disease pathways of the gut microbiota of HIV-infected patients.

## Highlights

• Alcohol use and gut microbial composition in PWH is an important area of research.
• Alcohol consumption alters the composition of the gut microflora in PWH.
• Alcohol consumption reduces metabolic pathways and cardiovascular disease pathways.

## Introduction

The Joint United Nations Programme on HIV/AIDS (UNAIDS) issued 90-90-90 treatment targets for HIV in 2014, and while some countries are still a significant way off from achieving this goal, good progress has been made overall (Granich et al., 2017). The use of antiretroviral therapy (ART) is effective in controlling HIV replication and extending the life expectancy of HIV-infected individuals (Legarth et al., 2016). However, HIV/AIDS patients have a high risk of multiple comorbidities through increased immune activation and inflammation compared to an uninfected population of the same age, including some cancers, diabetes, cardiovascular disease, and kidney disease (Ishizaka et al., 2021; Sarkar & Brown, 2021).

Alcohol abuse, the seventh leading cause of death and disability in humans (GBD 2016 Alcohol Collaborators, 2018), has the potential to lead to the development and worsening of several diseases, such as cardiovascular diseases, digestive diseases, cancers, and AIDS (Barberia-Latasa, Gea, & Martinez-Gonzalez, 2022; Chavez et al., 2021). Alcohol consumption is not uncommon in people with HIV (PWH) around the world, and sufficient evidence links alcohol consumption to an increased risk of HIV infection (Chavez et al., 2021; Mendez-Ruiz et al., 2020; Shuper, Joharchi, Monti, Loutfy, & Rehm, 2017). The frequency of high-risk sexual behavior increases after drinking alcohol, which in turn makes it more likely that HIV infection and transmission will occur. In addition, alcohol use and abuse decrease adherence to ART and also affect the effectiveness of ART in PWH (Baum et al., 2010; Chime, Ndibuagu, & Orji, 2019; Welsh et al., 2019). Alcohol can negatively influence the immune function of HIV-infected patients through various mechanisms (Bagby et al., 2015; Cook, Stapleton, Ballas, & Klinzman, 1997). Alcohol consumption was linked to decreased CD4+ T-cell counts regardless of the type of ART treatment PWH receives (Samet et al., 2007; Samet, Horton, Traphagen, Lyon, & Freedberg, 2003). Alcohol misuse was also related to elevated HIV viral loads among people on ART (Samet et al., 2003). More importantly, alcohol and its metabolites have been linked to increased physiological impairment and HIV mortality (Edelman et al., 2019; Justice et al., 2016). Non-hazardous alcohol consumption of one or more drinks per week reduced survival in PWH patients by 1 year, while patients with daily hazardous drinking had a 6.4-year reduction in survival (Braithwaite et al., 2007). PWH are usually advised to limit or avoid alcohol consumption by their doctors, because of the negative effects that alcohol may have on the immune system (Bagby et al., 2015; Barr, Helms, Grant, & Messaoudi, 2016).

There are more than 1000 microorganisms in the intestinal tract of healthy adults that encode genes that regulate human health and that are known as the "second genome" (Backhed, Ley, Sonnenburg, Peterson, & Gordon, 2005; Grice & Segre, 2012). The intestinal microbiota has an essential role in the immune system, and its ecological dysbiosis, that is, the disruption of the balance of the microbiota, has been shown to be related to a variety of diseases (Durack & Lynch, 2019; Ishizaka et al., 2021). Previous studies have shown that alterations in intestinal flora composition and compromised intestinal barrier integrity both occur in HIV-infected persons, and result in translocation of bacterial flora and their metabolites and a systemic inflammatory response (Marchetti, Tincati, & Silvestri, 2013; Mutlu et al., 2014; Ramendra et al., 2019). Like HIV, alcohol use and abuse have a pro-inflammatory effect, inducing dysbiosis, increasing intestinal permeability, and leading to microbial translocation (Gnatienko et al., 2018; Leclercq et al., 2012). Maffei and co-workers found that alcohol consumption was associated with CD8+ T-cell phenotype, intestinal leakage, and ecological dysregulation in PWH (Maffei et al., 2021). Alcohol-associated gut ecological dysregulation mediating susceptibility to pneumococcal pneumonia was reportedly observed in a mouse model of HIV (Samuelson et al., 2019). In addition, both animal models and human trials suggest that alcohol use and HIV may produce harmful synergistic reactions in the gut, and that alcohol consumption enhances HIV-induced intestinal damage (Banerjee, Abdelmegeed, Jang, & Song, 2015; Liu et al., 2019; Webel et al., 2017; Yan et al., 2021). The effects of alcohol misuse on gut microbiota are well known, but few studies have examined the relationship between alcohol consumption and gut microbiota in the specific population of HIV-infected individuals. We hypothesized that the composition of the gut microbiota is altered in HIV-infected individuals who consume alcohol compared to those who do not, and conducted this study.

## Materials and methods

### Study subjects and sample collection

Ninety-three PWH older than 18 years were recruited in Hefei, China, involving 21 low-to-moderate drinking HIV-infected participants and 72 non-drinking HIV-infected participants between November 2021 and May 2022. Study participants were recruited at Anhui Qingwei Public Health Service Center, a community service organization serving people affected by AIDS, as well as a national AIDS surveillance sentinel site and a health education base for HIV control and prevention in Hefei. Drinking ≥1 time/month or ≥12 times/year in the last year was defined as alcohol consumption. Basic information about the respondents was collected through a questionnaire, such as age, sexual orientation, CD4+ T-cell counts, and HIV-RNA values. Stools were collected by participants, and specimens were transported to the laboratory in an incubator within 4 h for dispensing, and then frozen at −80 °C for use at 6 months.

Exclusion criteria were as follows: 1) use of antibiotics, probiotics/prebiotics in the past 3 months; 2) diarrhea or gastrointestinal symptoms within the past 1 month; 3) presence of active opportunistic infections and co-infection with hepatitis B virus (HBV) and hepatitis C virus (HCV); 4) known to be pregnant or lactating; and (5) unwillingness to participate in this study.

### Calculation of alcohol consumption

Alcoholic beverages were divided into four categories: liquor, wine, rice wine, and beer. The total alcohol intake of each category of alcoholic beverages was calculated according to the weekly drinking frequency and the amount of alcohol consumed each time, and then according to the 2002 Chinese Food Composition Table – the amount of alcohol was calculated for each 100 g of alcoholic beverages: 43.1 g of liquor, 10.2 g of wine, 8.6 g of rice wine/fruit wine, and 4.3 g of beer. The total alcohol intake per person per week can be calculated by adding up the alcohol intake of the four types of alcoholic beverages (YG, 2014). Referring to the grouping status of alcohol intake in several drinking studies (low drinking: average total weekly intake of alcohol <70 g; moderate drinking: average total weekly intake of alcohol 70–210 g for men, average weekly intake of alcohol 70–140 g for women; excessive drinking: average weekly intake of alcohol ≥210 g for men, average weekly intake of alcohol ≥140 g for women) (Davies et al., 2002; O'Keefe, Bybee, & Lavie, 2007; Shaper & Wannamethee, 2000; Wannamethee & Shaper, 1997), it was calculated that all 21 individuals in the low-to-moderate drinking group consumed <210 g of alcohol per week.

### Genomics DNA extraction

Total DNA was collected from 200 to 300 mg of stool by magnetic bead purification technique using MagPure Stool DNA KF kit B (Magen, China). DNA sample concentrations were quantified using the Qubit ® dsDNA BR Detection Kit (Invitrogen, United States).

### DNA libraries and sequencing

The PCR reaction system (30 ng DNA sample, fusion PCR primers, and PCR master mix) was prepared strictly according to the kit instructions, and PCR amplification experiments were performed according to the operating procedures. Amplification of region V4 of bacterial 16S rDNA gene were done using degenerate PCR primers, 515F (GTGCCAGCMGCCGCGGTAA) and 806R (GGACTACHVGGGTWTCTAAT). The expanded products were purified and tested for library quality, and the libraries that passed the test were sequenced by Illumina MiSeq 2500 to generate sequencing libraries.

### Bioinformatics analysis

The raw data obtained from Illumina MiSeq 2500 sequencing were filtered to obtain clean data. The filtered clean data were spliced using the Fast Length Adjustment Program for Short Readers (FLASH, v1.2.11) to get tags for the high variation region (Magoc & Salzberg, 2011). UPARSE software (v7.0.1090) was used to cluster labels with 97% similarity of the spliced sequences into operational taxonomic units (OTUs) (Edgar, 2013). Representative sequences of OTUs were taxonomically annotated using the Ribosomal Database Project (RDP) classifier v.2.2 (compared to the RDP database: http://rdp.cme.msu.edu/).

The species accumulation curve was used to judge the sampling volume, and if there was a sharp increasing trend at the end of the curve, it indicated that the sampling volume was insufficient. Alpha diversity was assessed using Shannon and Chao-1 indexes by MOTHUR (v1.31.2) (Schloss et al., 2009), and principal coordinate analysis (PCoA) was run by QIIME (v1.8.0) (Caporaso et al., 2010). Permutational multivariate analysis of variance test was conducted usingnd weighted and unweighted UniFrac distances for beta diversity for comparison between groups. The Kyoto Encyclopedia of Genes and Genomes (KEGG) functions were predicted based on PICRUST2 (v2.3.0-b) to estimate microbial metabolic functions in this study (Wilkinson et al., 2018). Linear discriminant analysis (LDA) effect size (LEfSe) algorithm (https://huttenhower.sph.harvard.edu/galaxy/) was used to determine statistically significant bacterial taxa between groups. An LDA score >2.0 and *p* < 0.05 were considered statistically significant.

### Statistical analysis

R(v4.3.1) software was used to perform enteric flora data analysis. General demographic information of the participants was collected by questionnaire, organized, and entered into SPSS 23.0 software for data analysis. Data with non-normal distribution of CD4+ T-cell numbers and ART times are expressed using median and 4-digit spacing [M(P25, P75). The *t* test, *χ*^2^ test, or Mann–Whitney *U* test were used to compare basic demographic information between groups. Missing data were not included in the analysis. A two-tailed *p* value < 0.05 was considered statistically significant.

## Results

### General characteristics of the study population

**Fig. 1:**
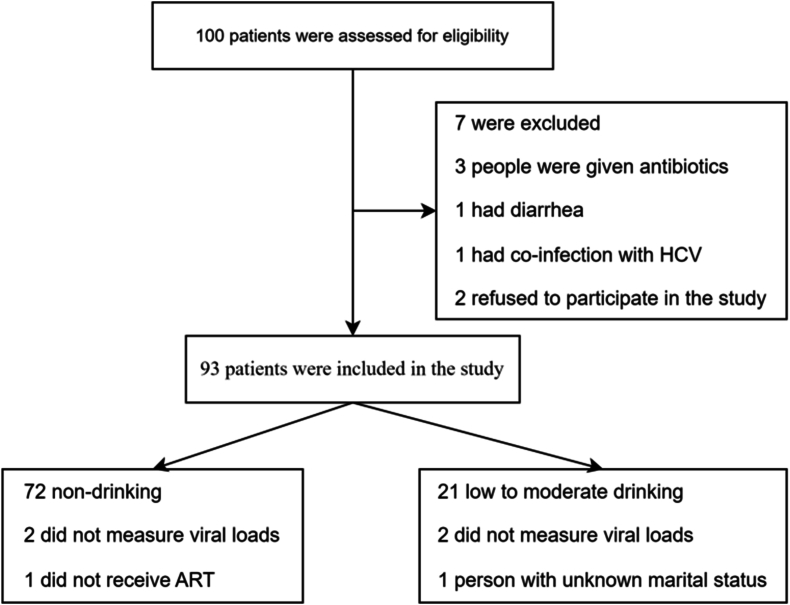
Flow diagram.

From November 2021 to May 2022, 100 HIV-infected patients were recruited at the Qingwei Public Health Service Center in Anhui, China. Seven cases were not included in the study, of whom 3 used antibiotics, 1 developed diarrhea, 1 was co-infected with HCV, and 2 declined to participate in the study (Fig. 1).

Ninety-three participants were divided into low-to-moderate drinking (<210 g of alcohol per week, n = 21) or non-drinking (n = 72) groups based on whether they consumed alcohol. A total of 4 of the 93 participants were not tested for viral loads, and 2 each were in the low-to-moderate drinking and non-drinking groups. All participants had been treated with ART except one in the non-drinking group. Smoking and alcohol consumption are often inseparable, and fortunately, there is no difference in smoking between the low-to-moderate drinking and non-drinking groups. The general characteristics between the low-to-moderate drinking and non-drinking groups were well balanced, as shown in Table 1. Dietary information was missing for two individuals in the non-drinking group, and dietary profiles were analyzed for the remaining 91 individuals. Except for "vegetables", which was statistically different between the two groups (*p* < 0.05), the rest of the diets were balanced between the two groups, and the results are presented in Supplementary Table 1. Moreover, all participants were able to supply stool specimens.

**Table 1:** General characteristics of the study population.

Characteristic	Non-drinking HIV-infected group (n = 72)	Low-to-moderate drinking HIV-infected group (n = 21) (<210 g of alcohol per week)	t/χ^2^/Z(95% CI)	*p* value
Age（year)	43.2 ± 11.1	42.1 ± 11.2	−0.410 (-6.628,4.362)	0.683
**Marital status**^a^				
No. (%) unmarried	28 (38.9)	10 (50.0)	3.572	0.168
No. (%) married/remarried	34 (47.2)	5 (25.0)		
No. (%) divorced/widower	10 (13.9)	5 (25.0)		
No. (%) males	67 (93.1)	21 (100.0)	−d	0.584
No. (%) of MSM^b^	53 (73.6)	19 (90.4)	1.768	0.184
No. (%) with viral loads ＜50 copies/mL	60 (85.7)	17 (89.5)	0.002	0.963
CD4 count (cells/μL)	399.5 (248.8, 548.0)	460.0 (281.5, 578.5)	−0.657^e^	0.511
Time on ART^c^ (year)	4 (2.0, 7.0)	4 (3.0, 8.0)	−0.785^e^	0.433
No. (%) of smokers	25 (34.7)	11 (52.4)	2.137	0.144

a One person's marital status unknown in low-to-moderate drinking group.

b MSM, men who have sex with men

c ART, antiretroviral therapy.

d Fisher's exact test.

e Mann-Whitney *U* test.

### 16s rDNA sequencing results

**Fig. 2:**
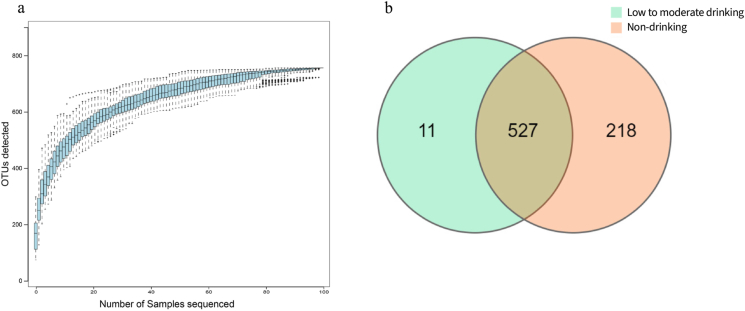
16s rDNA sequencing results. **a**) Species accumulation curves. **b**) OTU Venn diagram of between non-drinking and low-to-moderate drinking groups. **Blue**: low-to-moderate drinking HIV-infected group; **Pink**: non-drinking HIV-infected group.

As shown in Fig. 2a, the end of the curve tends to flatten out in the species accumulation curves, indicating that the sample size used for the study was adequate. The 16S rDNA sequencing results displayed 745 OTUs in the non-drinking group, including 527 OTUs shared with the low to moderate drinking group (Fig. 2b).

### Diversity analysis of gut microbiota

**Fig. 3:**
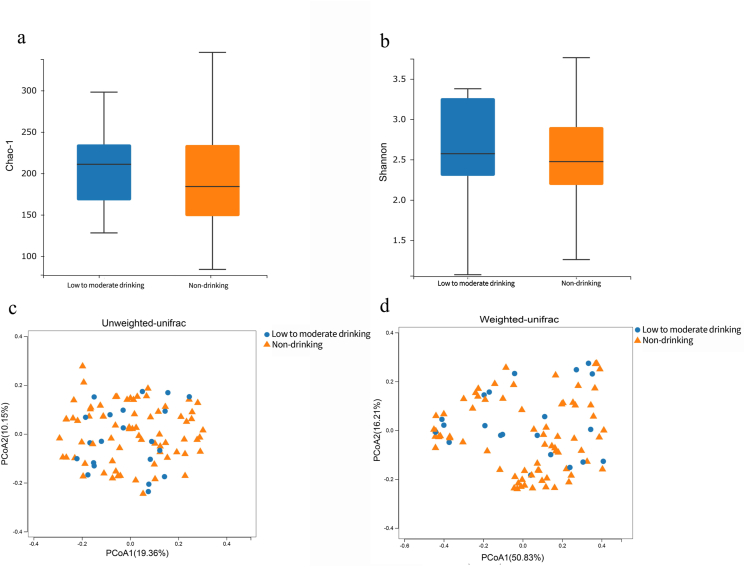
Comparison of intestinal flora diversity between the non-drinking and low-to-moderate drinking groups in HIV-infected individuals. **a** and **b**) Comparison of alpha diversity of intestinal microbiota between groups using Chao-1 and Shannon indices. **c** and **d**) Comparison of beta diversity of the intestinal microbiota as defined by principal coordinate analysis (PCoA). **Blue**: low-to-moderate drinking HIV-infected group; **Orange**: non-drinking HIV-infected group. (For interpretation of the references to color in this figure legend, the reader is referred to the Web version of this article).

The alpha diversity of the gut flora is reflected by the Shannon and Chao-1 indexes, which mainly reflect the community richness and evenness. The PCoA plot reflects the beta diversity of the intestinal flora and has been used to explore the similarities and differences in group composition. In this study, alpha diversity of the low-to-moderate drinking and non-drinking populations did not display statistically significant differences in Chao-1 (Fig. 3a, *p* = 0.323) and Shannon (Fig. 3b, *p* = 0.560) indices. Additionally, compared with controls, the fecal microbiota of the low-to-moderate drinking group did not show differences in beta diversity using the unweighted-unifrac (Fig. 3c, *p* = 0.744) and weighted-unifrac (Fig. 3d, *p* = 0.487). Due to the specificity of the methodology for analyzing gut flora, we were unable to control for vegetables as confounders during the analysis, with the exception of the alpha diversity analysis. After controlling for the frequency of vegetable use, we analyzed alpha diversity between groups and the differences were not statistically significant; see Supplementary Table 2. Overall, the intestinal microbiota diversity in HIV-infected individuals was not affected by low-to-moderate drinking.

### The gut microbiota was altered in the low-to-moderate drinking group

**Fig. 4:**
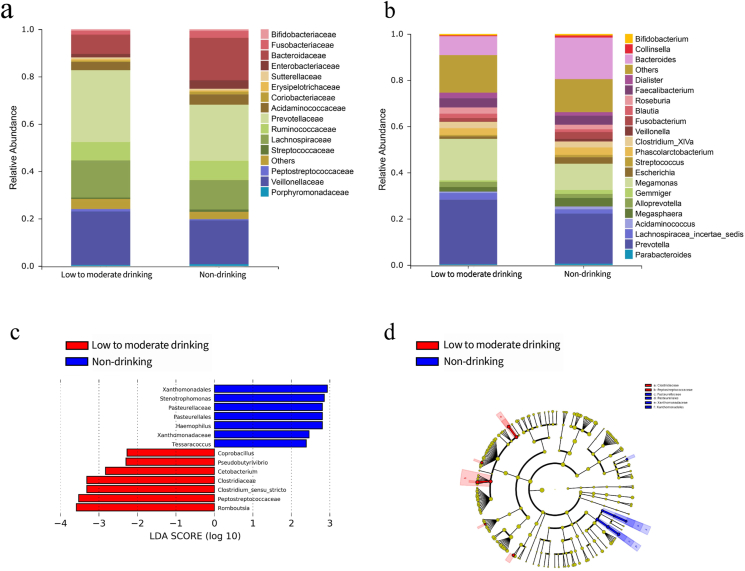
Fecal bacterial composition of the low-to-moderate drinking and non-drinking groups in HIV-infected patients. **a**) The relative abundance of gut microbiota at the family level. **b**) The relative abundance of gut microbiota at the genera level. **c**) Differentially enriched bacterial taxa were quantified as linear discriminant analysis effect sizes (LEfSe), showing taxa with linear discriminant analysis (LDA) scores greater than 2.0. **d**) Cladogram of LDA effect size. Species that were not annotated at this taxonomic level and whose abundance was less than 0.5% in the sample were combined into others. **Red**: low-to-moderate drinking HIV-infected group; **Blue**: non-drinking HIV-infected group. (For interpretation of the references to color in this figure legend, the reader is referred to the Web version of this article).

**Fig S1:**
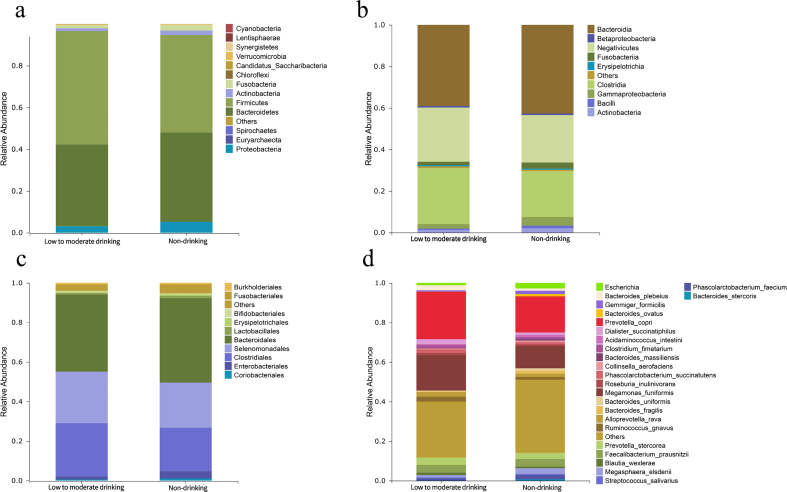
Fecal bacterial composition of the low-to-moderate drinking and non-drinking groups in HIV-infected patients. **a**) Phylum level. **b**) Class level. **c**) Order level. **d**) Species level. Species that were not annotated at this taxonomic level and whose abundance was less than 0.5% in the sample were combined into others.

Graphic representations were made of the relative proportions of taxa for each level in the between-group sample (Supplementary Fig. 1; Fig. 4a and b). More than 99% of the sequences in the analysis of taxonomic composition were classified into five phyla: *Firmicutes*, *Bacteroidete*, *Proteobacteria*, *Actinobacteria*, and *Fusobacteria* (Supplementary Fig. 1). At the family level, the top five families in terms of mean relative abundance among low-to-moderate drinking HIV-infected individuals were the same as in the non-drinking group; these five families were *Prevotellaceae*, *Bacteroidaceae*, *Lachnospiraceae*, and *Ruminococcaceae* (Fig. 4a). Compared to the non-drinking individuals, the low-to-moderate drinking individuals contained relatively abundant levels of *Prevotellaceae* (30.4% vs. 23.7%), *Lachnospiraceae* (15.7% vs. 12.4%), and *Veillonellaceae* (22.6% vs. 18.4%), and relatively lower levels of *Bacteroidaceae* (8.2% vs. 18.0%) and *Ruminococcaceae* (7.8% vs. 8.1%). However, the differences in relative abundance of the above five microorganisms were not statistically significant between groups (all *p* > 0.05). In addition, we observed an increase of *Megamonas* (*Firmicutes*) and *Prevotella* (*Bacteroidetes*) and a decrease of *Bacteroides* (*Bacteroidetes*) in the low-to-moderate drinking group (all *p* > 0.05) (Fig. 4b; Supplementary Fig. 1).

The LEfSe algorithm was used to compare the bacterial composition of the low-to-moderate drinking and non-drinking groups among HIV-infected individuals to identify specific gut microbes associated with alcohol consumption. LDA scores of differentially abundant taxa showed a higher representation of *Coprobacillus* (*Firmicutes*), *Pseudobutyrivibrio* (*Lachnospiraceae*), and *Clostridium*-sensu-stricto in the drinking group (Fig. 4c). The cladograms that were derived from the LEfSe analysis revealed that the low-to-moderate drinking group showed enrichment of *Clostridiaceae* and *Peptostreptococcaceae*, and depletion of *Pasteurellaceae* and *Xanthomonadaceae* (Fig. 4d).

### Functional microbiome profiles are altered in patients in the low-to-moderate drinking group

**Fig. 5:**
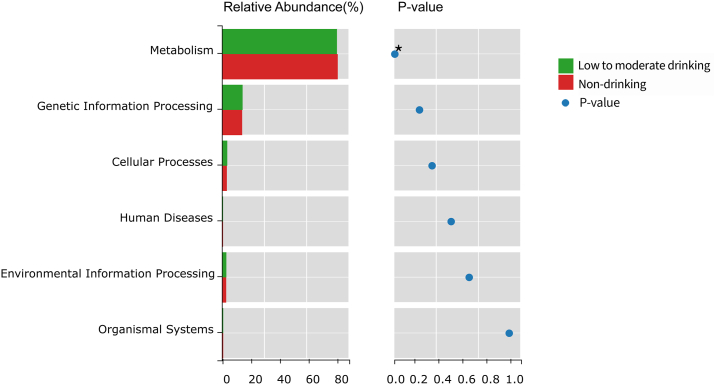
Prediction of functional of the intestinal microbiome using KEGG at level 1. ∗*p* = 0.036. **Green**: low-to-moderate drinking HIV-infected group; **Red**: non-drinking HIV-infected group; **Blue**: *p* value. (For interpretation of the references to color in this figure legend, the reader is referred to the Web version of this article).

To further characterize the gut microbiome link to low-to-moderate drinking, the KEGG pathway database was used to evaluate the functional profile in this research. There was a significant decrease in the metabolism pathways at level 1 (*p* = 0.036) (Fig. 5) and in the cardiovascular disease pathways at level 2 (*p* = 0.006) (Table 2) in the low-to-moderate drinking group.

**Table 2:** Prediction of functional of the intestinal microbiome using KEGG at level 2.^a^.

Function	Low-to-moderate drinking group (%)	Non-drinking group (%)	*p* value
Metabolism of cofactors and vitamins	13.742705	13.546001	0.416107
Cardiovascular diseases	2.60E-05	3.31E-04	0.005962
Cell growth and death	1.695313	1.613609	0.064096
Lipid metabolism	4.855478	5.262948	0.078472
Energy metabolism	5.27734	5.424279	0.108843
Translation	3.486575	3.380738	0.230483
Transport and catabolism	0.175142	0.18969	0.370311
Amino acid metabolism	12.990271	12.824991	0.121554
Glycan biosynthesis and metabolism	4.630141	5.028793	0.072435
Immune system	0.081654	0.081839	0.355769

a The remaining paths are not listed.

## Discussion

Currently, questions about the exact link between microbiome alterations and alcohol consumption during HIV infection are still to be answered. To clarify the relationships between altered microbiota composition and alcohol consumption in HIV-infected patients, we examined the diversity and composition of the fecal microbiota in low-to-moderate drinking and non-drinking patients. We found that low-to-moderate drinking decreased metabolism pathways and changed gut microbiota composition without affecting the variety of gut flora in HIV-infected individuals.

The results of the study showed enrichment of *Coprobacillus*, *Pseudobutyrivibrio*, *Lachnospiraceae*, *Peptostreptococcaceae*, and *Prevotella*, and depletion of *Pasteurellaceae* and *Xanthomonadaceae* in alcohol misuse HIV-infected populations. *Lachnospiraceae* is a potentially beneficial bacterium that can produce short-chain fatty acids (SCFAs). Studies have shown that low doses of alcohol may promote the release of anti-inflammatory fatty acids (e.g., SCFAs) (Bjorkhaug et al., 2019; Caslin, Mohler, Thiagarajan, & Melamed, 2021) and that alcohol itself is converted to SCFAs during metabolism (Caslin et al., 2021), revealing that alcohol, while affecting the gut microbial community, may positively impact gut health by modulating the production of SCFAs. In addition, butyric acid, a type of SCFA, is made by fermentation of fibers from intestinal bacteria and may improve intestinal barrier function (Russo et al., 2022; Wong, de Souza, Kendall, Emam, & Jenkins, 2006). Experiments conducted in animal models demonstrated that butyricnjected both intravenously and into the colon exhibited significant antihypertensive effects, and that the antihypertensive effects of injections into the colon were associated with a 2- to 3-fold increase in butyric acid levels in the colon (Onyszkiewicz et al., 2019). It is worth noting that hypertension is one of the major risk factors for cardiovascular disease (Vaduganathan, Mensah, Turco, Fuster, & Roth, 2022), and this study also found positive changes in cardiovascular disease pathways in the low-to-moderate drinking group. In addition, alcohol has a complex relationship with hypertension and cardiovascular disease, and may also reduce the risk of cardiovascular disease by increasing high-density lipoproteins and inhibiting the formation of blood clots (Kawano, 2010). Thus, the complex mechanisms linking alcohol consumption to hypertension and cardiovascular disease in HIV-infected individuals remain to be further investigated.

PWH have a higher prevalence of cardiovascular disease, their risk of developing cardiovascular disease is two times higher, and the global burden of associated disease is tripled (Ishizaka et al., 2021; Shah et al., 2018). HIV infection leads to a decrease in CD4+ T cells, disruption of the structure and function of the intestinal mucosal barrier, and an increased probability of opportunistic infections, thereby increasing the risk of cardiovascular disease (Marchetti et al., 2013; Mutlu et al., 2014; Ramendra et al., 2019; So-Armah et al., 2020). Efforts to answer questions about safe alcohol intake have been ongoing for many years, but controversy remains, and it is now generally accepted that the dose of alcohol consumed plays a vital role in the risk of developing cardiovascular disease. Some studies suggest that moderate-to-low dose alcohol consumption may be associated with protective effects against blood pressure, atrial fibrillation, and stroke due to the presence of substances with antioxidant properties in alcohol (Bazal et al., 2019; Fuchs & Fuchs, 2021; Haseeb, Alexander, & Baranchuk, 2017; Millwood et al., 2019). In addition, trimethylamine N-oxide (TMAO) is an intestinal microbe-derived metabolite that has been linked to cardiovascular (CV) function (Witkowski, Weeks, & Hazen, 2020). Elevated levels of TMAO have been associated with increased cardiovascular risk, contributing to atherosclerosis and adverse cardiovascular events (He, Tan, Xu, & Liu, 2020). More importantly, alcohol consumption has been shown to affect TMAO production (Coulbault et al., 2022). The complex relationship between the gut microbiota and its metabolites, alcohol use, and HIV still requires further research.

Studies have shown that the microbial composition of men who have sex with men (MSM) is characterized mainly by an increased abundance of *Prevotella*, whereas most non-MSM participants have an abundance of *Bacteroides*, which is not associated with HIV status (Armstrong et al., 2018; Noguera-Julian et al., 2016). In this study, the low-to-moderate drinking group also showed an accumulation of *Prevotella* and a decrease of *Bacteroides*, controlling for the sexual orientation of both groups of participants. Although the findings are similar, there is no consensus on whether the altered microbial composition in this population is due to sexual orientation or alcohol use, and further research is needed.

Improving intestinal dysbiosis may reduce microbial translocation and inflammation associated with alcohol consumption. Fecal microbiota transplantation, dietary interventions, and probiotic/prebiotic supplementation are often used to improve imbalances in the gut microbiome. Fecal microbiota transplantation and probiotic/prebiotic supplementation improve markers of intestinal damage and restore flora diversity (Cammarota et al., 2017; d'Ettorre et al., 2015; Serrano-Villar et al., 2017, 2021). Dietary interventions (e.g., vitamin A and D supplementation) may facilitate structural and functional recovery of the intestinal mucosal barrier (Cantorna, Snyder, & Arora, 2019). Additionally, zinc supplementation is a simple, cost-effective intervention that reduces the inflammatory response to continued alcohol consumption in HIV-infected patients (Gnatienko et al., 2018).

Our study had some limitations. First, there was a smaller sample size recruited in the low-to-moderate drinking HIV-infected group (only 21 people), and follow-up studies should expand the sample size. Second, although we calculated the amount of alcohol consumed by the survey respondents, we did not group them according to the amount of alcohol consumed because the sample size was too small. Thirdly, we only studied the relationship between gut microbial composition and alcohol consumption and did not perform tests for SCFAs and inflammatory factors, and therefore could not attempt to elucidate aspects such as microbial alterations leading to inflammation in the organism. Fourthly, the effect of smoking on intestinal flora is also a factor that cannot be ignored. Although there was no statistical difference in smoking between groups, the percentage difference was 17.7%, and subgroup analyses of smoking will be performed at a later stage when the sample size is expanded. Finally, participants in the low-to-moderate drinking group of this study consumed less than 210 g of alcohol per week and there was a lack of high-alcohol drinkers, and the frequency of vegetable use was also uneven between groups, so caution is needed in interpreting the findings of this study.

## Conclusion

In a study of alcohol consumption and gut microbiota in HIV-infected populations, we found an increase in SCFAs acid-producing bacteria, and a decrease in metabolic pathways and cardiovascular diseases pathways in the fecal microbiota of the low-to-moderate drinking population, with unchanged overall bacterial diversity. Future functional macrogenomics and metabolomics approaches could be applied to further research on the connection between alcohol consumption and gut microbes for the prevention of alcohol-related diseases in HIV-infected individuals.

## Ethics approval and consent to participate

The study was conducted in accordance with the Declaration of Helsinki of the World Medical Association, and the study protocol was approved by the Biomedical Ethics Committee of Anhui Medical University (approval number 20200594) with written informed consent obtained from all participants.

## Availability of data and materials

The datasets generated and/or analyzed during the current study are available in the Sequence Read Archive (SRA) repository [https://www.ncbi.nlm.nih.gov/sra/PRJNA957577].

## Funding

This work was supported by the IHM Center for Big Data and Population Health Research Fund (grant number JKS2022003), Chuzhou Municipal Health Commission Research Project (grant number CZWJ2022B002), and Inflammation and Immune Mediated Diseases Laboratory of Anhui Province Open Project (grant number IMMDL20220001).

## Authors' contributions

All authors participated in the design of the study. NNQ, QF, XHZ, and SSK performed the data sample collection, and NNQ, QF, ZWW, and GT processed the data and samples. NNQ and QF carried out the statistical analysis and composed the first draft. RXL and YGF gave opinions for modification. All authors read and approved the final manuscript.

## CRediT authorship contribution statement

**Ni-ni Qiao:** Writing – original draft, Investigation, Data curation, Conceptualization. **Quan Fang:** Software, Methodology, Investigation, Data curation, Conceptualization. **Su-su Ke:** Investigation, Data curation. **Xin-hong Zhang:** Investigation, Data curation. **Gan Tang:** Investigation, Data curation. **Zi-wei Wang**, Investigation, Data curation. **Yin-guang Fan:** Writing – review & editing, Supervision, Resources, Funding acquisition, Data curation, Conceptualization. **Rui- xue Leng:** Writing – review & editing, Supervision, Resources, Funding acquisition, Data curation, Conceptualization.

## Declaration of competing interest

The authors declare that they have no competing interests.
