# Supplementary material for: Effects of alcohol on the composition and metabolism of the intestinal microbiota among people with HIV: A cross-sectional study
Source: Alcohol. 2024 Nov;120:151–9. doi: 10.1016/j.alcohol.2024.02.003 (PMC11383188; doi:10.1016/j.alcohol.2024.02.003)
Supplement: Multimedia component 1 [file mmc1.docx]

**Supplementary Table 1.** Analysis of the dietary situation

| **Characteristic** | **Low-to-moderate drinking HIV-infected group (n=21)** | **Non-drinking HIV-infected group (n=70)** | ***χ*2** | ***p* value** |
| --- | --- | --- | --- | --- |
| **Regularity of diet** |  |  |  |  |
| Yes | 18（85.7） | 59（84.3） | 0.025 | 0.874 |
| No | 3（14.3） | 11（15.7） |  |  |
| **Coarse grains (maize, sorghum, etc.)** |  |  |  |  |
| None at all | 4（19.0） | 12（17.1） | 5.691^a^ | 0.215 |
| 1 time/month | 9（42.9） | 13（18.6） |  |  |
| 2-4 times/month | 4（19.0） | 26（37.1） |  |  |
| 2-3 times/week | 2（9.5） | 11（15.7） |  |  |
| ≥4 times/week | 2（9.5） | 8（11.4） |  |  |
| **Red meat** |  |  |  |  |
| None at all | 0（0.0） | 1（1.4） | 3.847^a^ | 0.447 |
| 1 time/month | 3（14.3） | 6（8.6） |  |  |
| 2-4 times/month | 2（9.5） | 11（15.7） |  |  |
| 2-3 times/week | 10（47.6） | 21（30.0） |  |  |
| ≥4 times/week | 6（28.6） | 31（44.3） |  |  |
| **Vegetables** |  |  |  |  |
| 1 time/month | 0（0.0） | 1（1.4） | 6.928^a^ | 0.041 |
| 2-4 times/month | 1（4.8） | 0（0.0） |  |  |
| 2-3 times/week | 8（38.1） | 13（18.6） |  |  |
| ≥4 times/week | 12（57.1） | 56（80.0） |  |  |
| **Fruits** |  |  |  |  |
| None at all | 1（4.8） | 13（4.3） | 5.715^a^ | 0.185 |
| 1 time/month | 1（4.8） | 13（4.3） |  |  |
| 2-4 times/month | 2（9.5） | 11（15.7） |  |  |
| 2-3 times/week | 12（57.1） | 21（30.0） |  |  |
| ≥4 times/week | 5（23.8） | 32（45.7） |  |  |
| **Fried and barbecued food** |  |  |  |  |
| None at all | 6（28.6） | 22（31.4） | 1.954^a^ | 0.77 |
| 1 time/month | 4（19.0） | 20（28.6） |  |  |
| 2-4 times/month | 6（28.6） | 15（21.4） |  |  |
| 2-3 times/week | 5（23.8） | 11（15.7） |  |  |
| ≥4 times/week | 0（0.0） | 2（2.9） |  |  |
| **Pickled food** |  |  |  |  |
| None at all | 7（33.3） | 10（14.3） | 5.486^a^ | 0.224 |
| 1 time/month | 5（23.8） | 16（22.9） |  |  |
| 2-4 times/month | 4（19.0） | 28（40.0） |  |  |
| 2-3 times/week | 5（23.8） | 14（20.0） |  |  |
| ≥4 times/week | 0（0.0） | 2（2.9） |  |  |
| **Yogurt** |  |  |  |  |
| None at all | 6（28.6） | 16（22.9） | 1.730^a^ | 0.823 |
| 1 time/month | 3（14.3） | 8（11.4） |  |  |
| 2-4 times/month | 4（19.0） | 20（28.6） |  |  |
| 2-3 times/week | 5（23.8） | 12（17.1） |  |  |
| ≥4 times/week | 3（14.3） | 14（20.0） |  |  |
| **Tea** |  |  |  |  |
| None at all | 7（33.3） | 22（31.4） | 1.658^a^ | 0.831 |
| 1 time/month | 1（4.8） | 9（12.9） |  |  |
| 2-4 times/month | 3（14.3） | 7（10.0） |  |  |
| 2-3 times/week | 2（9.5） | 10（14.3） |  |  |
| ≥4 times/week | 8（38.1） | 22（31.4） |  |  |

^a^Fisher's exact test
