# Supplementary material for: Effects of alcohol on the composition and metabolism of the intestinal microbiota among people with HIV: A cross-sectional study
Source: Alcohol. 2024 Nov;120:151–9. doi: 10.1016/j.alcohol.2024.02.003 (PMC11383188; doi:10.1016/j.alcohol.2024.02.003)
Supplement: Multimedia component 2 [file mmc2.docx]

**Supplementary Table 2A.** Generalized linear model analysis of intestinal flora alpha diversity (chao-1 index)

|  | *β* | 95% CI | | χ^2^ | *p* value |
| --- | --- | --- | --- | --- | --- |
|  |  | Lower | Upper |  |  |
| Low-to-moderate drinking（Reference: non-drinking） | 14.527 | -15.591 | 44.645 | 0.894 | 0.344 |
| Vegetables | 10.812 | -12.671 | 34.295 | 0.814 | 0.367 |

**Supplementary Table 2B.** Generalized linear model analysis of intestinal flora alpha diversity (Shannon index)

|  | *β* | 95% CI | | χ^2^ | *p* value |
| --- | --- | --- | --- | --- | --- |
|  |  | Lower | Upper |  |  |
| Low-to-moderate drinking（Reference: non-drinking） | 0.049 | -0.265 | 0.363 | 0.094 | 0.759 |
| Vegetables | 0.174 | -0.071 | 0.419 | 1.94 | 0.164 |
